# Follicle on the Roof: Tertiary Lymphoid Structures in Central Nervous System Autoimmunity

**DOI:** 10.1111/imr.70045

**Published:** 2025-06-26

**Authors:** Michelle Zuo, Angela A. Wang, Jennifer L. Gommerman

**Affiliations:** ^1^ Department of Immunology University of Toronto Toronto Ontario Canada

**Keywords:** autoimmunity, B cells, experimental autoimmune encephalomyelitis, lymphotoxin, multiple sclerosis, neutrophils, Th17

## Abstract

Leptomeningeal tertiary lymphoid structures (TLS) have emerged as a relatively common pathological feature of autoimmune disease, including multiple sclerosis (MS) and particularly in people with progressive and nonremitting MS. These ectopic lymphoid aggregates, observed in the leptomeninges adjacent to so‐called “Type 3” sub‐pial cortical lesions, are associated with more severe gray matter damage and worse clinical outcomes. Mouse models of MS that recapitulate TLS formation in the central nervous system (CNS) have provided critical insights into the mechanisms driving their development and maintenance. In these models of experimental autoimmune encephalomyelitis (EAE) initiation of TLS is facilitated by Th17 cells, which promote chronic inflammation via cytokines such as IL‐17 and GM‐CSF. The cell surface expression of lymphotoxin‐α and lymphotoxin‐β heterotrimers (LTαβ) on lymphocytes, including Th17 cells, elaborates the organization of ectopic lymphoid tissues via LTβR signaling on radio‐resistant stromal cells and resident fibroblasts. Ultimately a pro‐inflammatory environment characterized by cytokines such as IL‐17 and GM‐CSF promotes the recruitment of neutrophils which produce proteases and chemokines that sustain a pro‐inflammatory milieu. Emerging EAE data suggest that disrupting TLS organization or targeting key pathways involved in their maintenance could represent promising strategies for modulating chronic CNS inflammation in MS. Understanding the cellular and molecular mechanisms regulating TLS dynamics is therefore critical for the development of therapies aimed at halting or reversing nonremitting MS disease.

## 1 Introduction

Adaptive immune responses are initiated in secondary lymphoid organs, such as the spleen and regional lymph nodes. The organization of lymphocytes within secondary lymphoid tissues is key to generating an efficient adaptive immune response. Development of secondary lymphoid organs begins during embryogenesis, where fetal liver‐derived hematopoietic lymphoid tissue inducer (LTi) cells interact with mesenchymal lymphoid tissue organizer (LTo) cells at future sites of lymphoid organ development [1]. Lymphotoxin‐αβ (LTαβ), produced by LTi cells, promotes the production of chemokines and expression of adhesion molecules on LTos through LTβR signaling. Consequently, the LT pathway is necessary for secondary lymphoid organ development [1]. Such developmental LTβR‐dependent signals are echoed in the adult animal during homeostasis to maintain the chemokine networks that are essential for the organization of lymphocytes in secondary lymphoid organs [2, 3], and can drive the formation of so‐called tertiary lymphoid structure (TLS) in chronically inflamed tissues [4]. TLS, which resemble secondary lymphoid tissues insofar as they contain aggregates of T cells, B cells, and antigen‐presenting cells (APCs) supported by fibroblasts that produce a network of extracellular matrix (ECM), have been reported in the context of cancer [5], autoimmunity [6], transplantation [7, 8], and infection [9]. TLS is observed across multiple tissues, including a compartment adjacent to the central nervous system (CNS) called the leptomeninges [10]. In contrast to TLS in other tissues and pathologies, such as those observed in the salivary glands of Sjogren's disease patients [11] or the joints of rheumatoid arthritis patients [12], T cell and B cell zones in leptomeningeal TLS are less defined and evidence for bona fide GC reactions is lacking [13, 14]. Nevertheless, leptomeningeal TLS has garnered special attention in multiple sclerosis (MS) research as its presence has been correlated with clinical measures of disease progression [15]. For the purposes of this review, sites of leptomeningeal immune cell aggregates containing T cells and B cells will be considered TLS. We will highlight findings on leptomeningeal TLS formation, persistence, and function in MS and its animal model, experimental autoimmune encephalomyelitis (EAE), then discuss evidence that may rationalize the disruption of TLS as a therapeutic strategy for attenuating MS progression.

## 2 Anatomy of the CNS and Meningeal Layers

**FIGURE 1:**
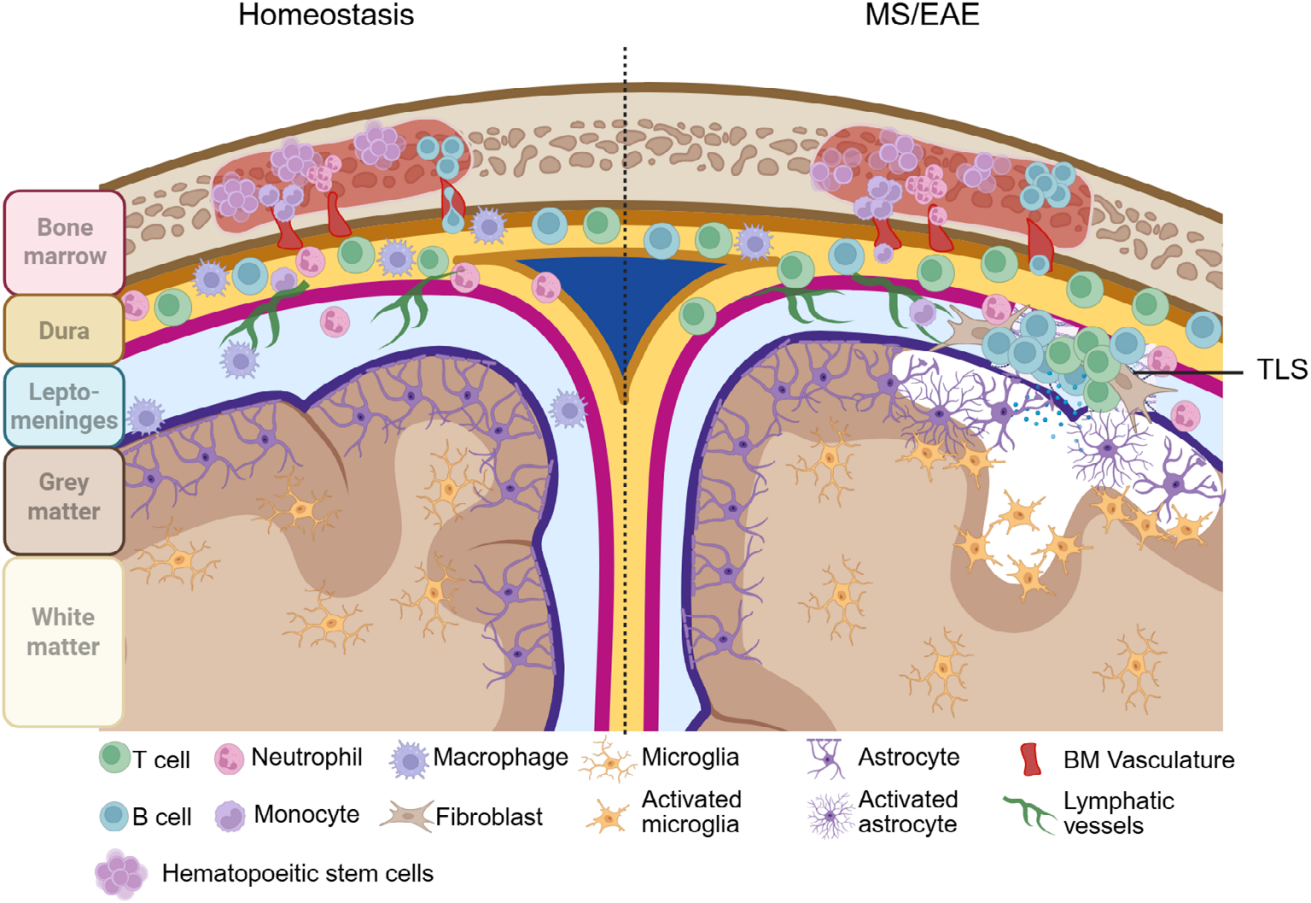
Structure of the steady‐state and inflamed meninges and brain. (Left) Homeostatic CNS and meninges: Reservoir of immune cells in the dura are continually supplied by the skull bone marrow, while leptomeninges remain relatively clear of immune cells and toxic factors. Lymphatic vessels in the leptomeninges drain solutes from CSF into dural sinuses. Underlying glia limitans maintained by tight junctions between astrocyte end feet are intact, and gray and white matter are healthy and myelinated. Microglia remain in resting state. (Right) Pathogenic T cells induce elaboration of fibroblast niche, recruitment of neutrophils and B cells from the periphery, and production of toxic factors that disrupt the glia limitans. A combination of noxious solutes from the leptomeninges and activated microglia leads to subpial gray matter damage (lesion). Persistent gray matter damage eventually results in neuronal death and cortical atrophy (not shown).

The meninges are composed of three distinct layers that envelop and protect the brain. The innermost layer, the pia mater, is in direct contact with the underlying brain parenchyma. Above it, the arachnoid mater is connected to the pia by fine connective tissue strands called trabeculae. The space between these two layers—the subarachnoid space (SAS), also referred to as the leptomeninges—is filled with cerebrospinal fluid (CSF). Overlying the arachnoid is the dura mater, a dense, fibrous layer that secures the meninges to the skull (Figure 1).

While small numbers of T lymphocytes reside in the CNS, presumably for the purpose of immunosurveillance [16, 17, 18], for the most part, the brain and spinal cord parenchyma are largely devoid of T cells and B cells at homeostasis. This paucity in lymphocytes is in part owing to tight endothelial junctions in the blood–brain barrier (BBB) which limits blood‐derived cells and proteins from entering the CNS [19]. However, unlike the tightly regulated vasculature of the blood–brain barrier, dural blood vessels are fenestrated and lack tight junctions, permitting the passage of large molecules and peripheral immune cells into this meningeal compartment [20, 21]. For this reason, the dura is densely populated by immune cells including lymphocytes, monocytes, and other myeloid cells, even under steady‐state conditions [20]. The anatomical and immunological features of the dura mater are quite unique. Adhered to the inner surface of the skull, the dura contains specialized structures known as dural venous sinuses, which facilitate the drainage of venous blood from the brain. In addition to venous channels, the dura is also traversed by arteries derived from the carotid circulation and veins that ultimately drain into the dural sinuses [20]. Dural blood vessels are also innervated and responsive to neurovascular signaling, allowing for dynamic interactions between the nervous and immune systems. Another unique feature of the dura is its connectivity to the skull bone marrow via diploic veins, which offer a direct migratory route for immune cells [20]. For example, in a mouse model of stroke, neutrophils were observed migrating from the skull bone marrow into the dura, underscoring the potential functional relevance of this tissue during injury and/or inflammation [22]. Furthermore, the calvarial bone marrow harbors distinct niches that communicate with both the dura and the SAS through osseous channels, providing a potential source of immune cells during CNS inflammation. A pivotal study by Marco Colonna's group showed that dural B cells originate from the skull bone marrow and migrate into the dura through skull vascular channels potentially in response to a gradient of CXCL12 chemokine [23]. Using an intrathecal injection of CD19‐tdTomato cells to track migration, Colonna and colleagues found tdTomato^+^ B cells in both dural lymphatic vessels and cervical lymph nodes 24 h following transfer, suggesting that the dura may be a migratory route for B cells [23]. Ongoing research continues to explore the role of skull bone marrow in regulating immune responses in the context of neuroinflammatory disease [23, 24].

The arachnoid mater at the base of the dura consists of squamous epithelial cells joined by tight junctions and is supported by a meshwork of collagenous trabeculae that span the SAS, forming the leptomeninges through which CSF circulates. The presence of tight junctions within the arachnoid epithelium establishes a physiological barrier between the dura mater and the CSF‐filled SAS. In addition to its structural role, the arachnoid epithelium expresses various efflux drug transporters and cytochrome p450 enzymes [25], suggesting it plays an active role in regulating molecular clearance from the CSF. Beneath the arachnoid lies the pia mater, a thin, delicate membrane that closely follows the contours of the brain and serves as a barrier between the parenchyma and penetrating blood vessels. Immediately below the pia is the glia limitans, a continuous layer of astrocyte end‐feet that forms the final boundary of the blood–meningeal barrier (BMB) [26, 27]. This multilayered system functions collectively to regulate immune cell access to the brain and maintain CNS immune privilege under homeostatic conditions.

In contrast to the fenestrated vasculature of the dura mater, blood vessels within the leptomeninges are nonfenestrated and sealed by tight junctions, forming a selective barrier that limits the extravasation of immune cells and macromolecules from the circulation into the CSF [26, 28]. This barrier is maintained by tightly connected endothelial cells and by bidirectional crosstalk with astrocytes of the glia limitans, which reinforces barrier integrity. A defining feature of these astrocytes is the expression of aquaporin‐4 (AQP4), a water channel protein enriched on astrocytic end‐feet surrounding cerebral blood vessels [29]. In vitro coculture models of the blood–brain barrier (BBB) have shown that astrocytes play a critical role in modulating endothelial expression of tight junction proteins, emphasizing the importance of astrocyte–endothelial cell cooperation in maintaining barrier function [30]. Despite the restrictive nature of the leptomeningeal vasculature, low‐level immune cell trafficking does occur under homeostatic conditions. Endothelial expression of adhesion molecules such as P‐selectin, E‐selectin, and intercellular adhesion molecule‐1 (ICAM‐1) supports limited leukocyte surveillance of the leptomeningeal compartment [31]. Additionally, C‐C chemokine ligand 19 (CCL19) is constitutively present in human CSF and may contribute to the basal recruitment of CCR7‐expressing leukocytes, even in the absence of inflammation [32].

There is debate in the literature regarding the presence and continuity of perivascular spaces within the SAS and cerebral cortex, and whether these are structurally or functionally continuous with Virchow‐Robin (VR) spaces, which envelop parenchymal penetrating blood vessels in the white matter. While VR spaces in the white matter are increasingly recognized as sites of immune surveillance and potential antigen presentation, the existence of analogous perivascular compartments surrounding leptomeningeal or cortical vessels is less well defined. However, if present, these perivascular spaces could offer a transient niche for immune cell trafficking and retention within the SAS, supporting localized immune surveillance even under noninflammatory conditions [33, 34, 35, 36]. In support of the concept that immune cells occupy the leptomeninges, flow cytometric studies of human CSF have revealed the presence of memory CD4^+^ T cells and occasional B cells under steady‐state conditions [31, 37]. Correspondingly, murine histological studies have identified a diverse population of immune cells within the meninges, including macrophages, dendritic cells, neutrophils, and mast cells [31, 38, 39, 40]. Recent transcriptomic analyses have further confirmed the presence of natural killer (NK) cells, T cells, and B cells in the leptomeninges of mice [38]. These findings suggest that, like the dura, the leptomeninges maintain a resident or semi‐resident immune cell population. However, the duration of immune cell residency within the SAS and their functional dynamics remains poorly defined. What is clear is that the density and diversity of immune cells in the leptomeninges are likely more limited than the dura, which appears to be more immunologically active at baseline.

Although relatively lymphocyte‐poor in steady state, the leptomeninges can alter dramatically during inflammation. This may be due to “hair‐trigger”‐like changes in resident fibroblast populations within the SAS. Fibroblasts are specialized stromal cells that are important for more than just architectural integrity of a tissue. In peripheral tissues there are a variety of fibroblasts that respond to inflammation, and lessons from these fibroblasts may give some insights into meningeal fibroblast remodeling [41, 42]. For example, Buechler et al. provided a framework for identifying and functionally characterizing fibroblasts, outlining that while there is heterogeneity of fibroblast populations within tissues, they adopt similar functional states in response to inflammation [42]. Inflammatory fibroblasts express conserved genes across tissues, including those involved in cytokine signaling (*Il6*, *Ccl2*, *Cxcl1*), ECM remodeling (*Mmp3*, *Mmp13*), and immune cell recruitment (*Cxcl12*, *Ccl19*, *Ccl21*) [42].

Although our understanding of fibroblasts in the leptomeninges is in its infancy, we previously characterized a network of CD31^−^ podoplanin (PDPN)^+^ fibroblastic reticular cell (FRC)‐like stromal cells in the leptomeninges in mice [43, 44]. FRC organizes immune cell positioning and facilitates immune responses in secondary lymphoid organs via the production of various chemokines and survival factors [44, 45]. Experimental models of CNS infection—including viral and parasitic pathogens such as *Toxoplasma gondii*—demonstrate that leptomeningeal fibroblasts can upregulate chemokines such as CCL21, enabling the recruitment of CD8^+^ T cells into the SAS for pathogen clearance [46, 47]. As will be discussed in a later section, evidence from animal models of MS (EAE) has shown that fibroblasts are key orchestrators of leptomeningeal TLS.

## 3 Leptomeningeal TLS in MS

MS is widely regarded as an autoimmune disease directed against myelin that ensheathes axons, manifesting as neurological symptoms such as cognitive deficits and motor dysfunction [48]. Clinically, MS is classified into three subtypes: relapse–remitting MS (RRMS), primary progressive MS (PPMS), and secondary progressive MS (SPMS) [49]. Many people are initially diagnosed with relapsing–remitting MS, where acute symptoms are followed by a time of relative quiescence. With time, individuals may transition into progressive MS (PMS), where the influx of lymphocytes into the CNS has subsided and neurodegenerative processes dominate [50]. However, emerging evidence suggests that MS may invoke parallel disease processes that occur from disease onset [51]. MS diagnosis and monitoring of progression is achieved by longitudinal assessment of clinical deficits, using metrics such as the Expanded Disability Scoring System or white matter lesion burden using magnetic resonance imaging (MRI) [48]. Neither of these metrics clearly distinguishes between RRMS and PMS.

What triggers MS is unclear, but a genetic component is evident. Widescale genome mapping studies across different populations have consistently implicated a link between MS and *HLA‐DRB1* risk alleles which encode for MHC‐II [52, 53]. Genetic variations in HLA genes determine the repertoire of peptides presented by MHC molecules, which in turn shapes CD4^+^ T cell reactivity and their capacity to provide help to cognate B cells. MS patient‐derived blood samples show evidence of elevated systemic T cell activation [54], impaired regulatory T cell function [55], and T cell autoreactivity against myelin‐derived peptides, highlighting the critical involvement of T cell dysregulation in disease. CD4^+^ T cells are thus thought to play a central role in MS pathogenesis, a concept also strongly supported by animal model data [56, 57]. The presence of oligoclonal antibody bands (OCBs) in MS CSF is evidence of abnormal intrathecal antibody production. This prompted the testing of B cell–depleting therapies in MS, such as anti‐CD20 antibodies (rituximab, ocrelizumab) [58]. These therapies have a profound impact on reducing relapsing biology in MS; however, this benefit occurs without altering levels of CSF oligoclonal bands [59]. One hypothesis is that the mechanism of action of anti‐CD20 antibodies in reducing relapsing biology may be linked to antibody‐independent B cell functions, such as production of anti‐inflammatory cytokines or T cell inhibitory ligands [60, 61].

The mechanisms underlying the transition from RRMS to PMS remain incompletely understood. One proposed explanation for this shift involves the gradual accumulation of gray matter (GM) injury [62, 63, 64]. Both imaging and histopathological studies have demonstrated the presence of GM abnormalities early in the disease course, with lesion burden intensifying as the disease advances [64, 65, 66]. Multiple cross‐sectional and longitudinal investigations have further established a correlation between GM pathology and the progression of physical and cognitive impairments [66, 67, 68]. Notably, the volume of cortical GM lesions has emerged as a robust predictor of disease progression and an indicator of the transition from RRMS to PMS. While various types of GM lesions exist, this review focuses on subpial cortical lesions, also called Type III lesions, which have been documented in postmortem MS brain tissue [65]. Type III lesions are localized to the surface of the brain, frequently forming ribbon‐like patterns across multiple gyri. Importantly, such lesions are not observed in other inflammatory CNS diseases such as Rasmussen's encephalitis [69] or neuromyelitis optica (NMO) [70], suggesting they are unique to MS [63]. Beyond demyelination, these lesions are also characterized by axonal, neuronal, and synaptic injury/loss [62, 63].

A hallmark of Type III lesions is their spatial association with leptomeningeal TLS [14, 15, 71]. Leptomeningeal TLS in postmortem MS brain tissues has been shown to contain a variety of immune cell types, including B cells, T cells, dendritic cells, macrophages, plasma cells, and stromal cells resembling follicular dendritic cells [14, 15, 72]. The presence of CXCL13 within these structures supports the recruitment and retention of CXCR5^+^ lymphocytes, including B cells and likely T follicular helper (Tfh) cells [73]. Moreover, RORγt^+^ Th17 cells, which can produce IL‐17, are preferentially enriched within immune aggregates [74] rather than diffusely infiltrated meningeal areas, suggesting that they play a role in the organized formation of these tertiary lymphoid‐like structures.

As mentioned, the glial limitans form a tight barrier reinforced by astrocyte end‐feet processes that separate the leptomeninges from the CNS parenchyma [45]. Due to this barrier, it is unlikely that leptomeningeal TLS‐resident immune cells directly penetrate the underlying cortex during MS/EAE. Instead, it is hypothesized that diffusible inflammatory mediators—such as cytokines, chemokines, and other toxic molecules—cross the glia limitans and induce localized subpial damage leading to Type III lesions. Supporting this model, CSF from people with MS with high cortical lesion burden exhibits elevated levels of proinflammatory cytokines (e.g., IFN‐γ, TNF, IL‐2, IL‐22), lymphoid‐organizing factors (e.g., CXCL13, LTα), B cell survival factors (e.g., BAFF), molecules associated with BBB dysfunction (e.g., fibrin, complement, coagulation proteins), and iron‐related oxidative stress indicators (e.g., hemoglobin, haptoglobin) [75, 76, 77].

Although subpial GM lesions are anatomically distinct from white matter (WM) lesions, there may be a connection [78]. Recent work revealed that postmortem tissues from people with MS that had high meningeal T and B cell infiltration exhibit not only more extensive subpial demyelination but also a higher frequency of active and mixed active‐inactive WM lesions relative to inactive or remyelinated WM lesions [78]. This observation aligns with longitudinal MRI studies linking WM lesion volume to GM atrophy [79, 80]. While it is not known how leptomeningeal inflammation is connected to WM lesions, these data suggest a broader interplay between CNS‐compartmentalized inflammation in the leptomeninges and widespread CNS pathology [69]. One possibility is that antigens released from chronically inflamed WM lesions drain via dural lymphatics to the cervical lymph nodes where they prime autoreactive T cells that home back to the leptomeninges [78]. Alternatively, axonal degeneration within subcortical WM may initiate retrograde damage to cortical neurons, leading to secondary GM injury [78].

While postmortem studies have been instrumental in characterizing the cellular and molecular composition of leptomeningeal TLS, translating these findings to living individuals remains difficult. Advanced imaging techniques, such as high‐resolution MRI with contrast enhancement, have provided some evidence of leptomeningeal contrast dye enhancement [81, 82, 83], yet these measurements are unable to confirm that these areas of contrast are due to resident lymphocytes versus residual fibrotic scarring. Moreover, the development of consistent and sensitive biomarkers to reflect leptomeningeal inflammation is hindered by the compartmentalized nature of the immune response, which may not be adequately captured by peripheral blood or even CSF analyses. Although certain cytokines and chemokines such as CXCL13, BAFF, and LTα have been associated with leptomeningeal TLS and cortical pathology [84], their levels can vary widely between individuals and disease stages, limiting their clinical utility. These technical and biological limitations underscore the need for novel, multimodal approaches to detect and monitor leptomeningeal TLS in vivo and to establish reliable biomarkers that can guide prognosis and therapeutic intervention, as well as animal models that replicate the relationship between leptomeningeal TLS and Type III sub‐pial lesions.

## 4 Mouse Models of Leptomeningeal TLS in MS

EAE is the collective term for animal models that mimic characteristics of MS, particularly CD4^+^ T cell‐mediated demyelination. The earliest deliberate induction of what is now known as EAE was established by Koritschoner and Schweinburg through the injection of human spinal cord homogenates into rabbits [85]. It was then shown in rhesus monkeys that the paralysis seen after repeated brain homogenate injections was accompanied by demyelinating brain lesions [86] Subsequently, EAE has been induced across a variety of model organisms and leveraged as a model for MS [87, 88, 89]. Amongst these, mouse models are the most widely used, benefiting from the availability of genetically modified strains that provide powerful tools for neuroimmunology research. Mouse models of EAE rely on sensitizing the adaptive immune system towards myelin proteins or peptides, such as myelin oligodendrocyte protein (MOG), proteolipid protein (PLP), and myelin basic protein (MBP), which can lead to different disease courses depending on the route of administration and the mouse strain used [90]. In active EAE models, mice are immunized towards myelin peptides or proteins, usually emulsified in Complete Freund's Adjuvant (CFA) which contains heat‐killed *Mycobacterium tuberculosis* [90]. This generates an encephalitogenic CD4^+^ T cell response towards a dominant myelin epitope and drives demyelination in a manner that, depending on the immunogen, may or may not depend on B cells [91, 92, 93]. Alternatively, passive EAE models involve the adoptive transfer of pre‐primed encephalitogenic CD4^+^ T cells into a naïve recipient animal, which provides an opportunity to study CD4^+^ T cell‐driven effector function independent of priming events [94, 95]. Spontaneous EAE models also exist—these are mice genetically engineered to express a T cell receptor and B cell receptor transgenes specific for myelin [94]. Ultimately, mouse EAE variations seek to recapitulate specific pathological features of disease, including a relapsing–remitting versus progressive disease course, and the localization of leptomeningeal TLS (Table 1).

### 4.1 C57Bl/6 EAE

MOG_35‐55_ EAE in C57Bl/6 mice is the most widely used EAE model for its robust CNS‐directed autoimmune CD4^+^ T cell response and the availability of genetically modified mice. Active MOG_35‐55_ EAE requires coadministration of pertussis toxin, which interrupts G protein‐coupled receptor signaling and has been reported to transiently loosen the BBB [108, 109]. C57Bl/6 mice typically develop a disease typified by acute onset followed by some extent of recovery and subsequent chronic disease [95, 108, 110]. Spinal cord inflammation dominates this model, with leptomeningeal TLS rarely reported in the brain [111]. One study has reported that stereotactic injection of heat‐killed *Mycobacterium tuberculosis* into the piriform cortex after immunization with MOG_35‐55_ is capable of inducing focal lesions in the brain, which are termed delayed‐type hypersensitivity (DTH)‐TLS [97]. In this model, T cell and B cell infiltration into the leptomeninges increases over time and is accompanied by demyelination, as well as microglial and astrocyte activation proximal to overlying leptomeningeal aggregates [97]. For these reasons, DTH‐TLS has been proposed as a model for recapitulating leptomeningeal TLS‐associated pathology observed in PMS.

TLS in the spinal cord leptomeninges in C57Bl/6 mice has mostly been studied in the context of transgenic myelin‐specific TCR and BCR mice. T cells derived from mice that express a transgene encoding a MOG_35‐55_‐specific TCR composed of the Vα3.2 and Vβ11 chains (2D2 TCR) [98] are capable of inducing EAE in naïve WT C57Bl/6 recipient mice upon adoptive transfer. These T cells induce TLS in the spinal cord SAS that vary in size but are comprised of B cell clusters surrounded by T cells and encapsulated within reticulin^+^ ECM [112]. A MOG‐specific IgH (IgH^MOG^) knock‐in has also been generated [99]. Both 2D2 and IgH^MOG^ mice are individually susceptible to EAE following immunization with MOG peptides [98, 113], or full‐length MOG protein [98]. When crossed together, 2D2 x IgH^MOG^ mice develop spontaneous EAE characterized by B cell aggregates in the spinal cord leptomeninges [114, 115, 116]. These leptomeningeal TLS‐resident B cells are CD62L^low^ and CD80^high^, suggesting previous activation or priming. However, unlike secondary lymphoid tissues, these TLS do not contain distinct T cell and B cell zones nor do they support germinal centers [116]. Single‐cell transcriptomic analyses of leptomeningeal TLS from 2D2 × IgH^MOG^ mice identified clusters of cells resembling follicular and marginal zone B cells, as well as various populations of CD4^+^ T cells and myeloid cells—lymphocyte populations reminiscent of their counterparts in the spleen and lymph nodes, albeit leptomeningeal lymphocytes exhibit a more proinflammatory phenotype [117].

### 4.2 Nonobese Diabetic (NOD) EAE

NOD mice have traditionally been used as a model for type 1 diabetes (T1D) for their spontaneous onset of disease driven by an autoimmune T cell response towards pancreatic beta cell antigens [100]. However, NOD mice are also susceptible to EAE upon immunization with MOG_35‐55_ [101]. Immunized mice initially develop relapse‐remitting EAE which transitions into progressive disease worsening [118], although there is some debate as to whether this resembles true progression as seen in people with MS [119]. Nonetheless, the model has been successfully used to study axonal injury [120], astrocyte activation [118, 121], and mechanisms leading to cortical demyelinating lesions [122]. One study reported the development of TLS in the spinal cord leptomeninges of chronic NOD‐EAE mice that were populated by CD4^+^ T cells, B220^+^ B cells, and CD21/CD35^+^ follicular dendritic cells (FDCs) [102]. Additionally, transcriptomic analysis of chronic NOD‐EAE spinal cords showed evidence of stromal cell remodeling, revealing a potential mechanism for TLS formation in this model [102].

To study the role of myelin‐reactive B cells, transgenic IgH^MOG^ mice were backcrossed onto the NOD background [123]. Unlike NOD WT mice, which present initially with relapsing–remitting disease, NOD IgH^[MOG]^ mice develop a rapid and severe form of progressive EAE after active immunization with MOG_35‐55_ peptide [103]. In this model, brain‐adjacent leptomeningeal TLS are observed and are colocalized with a fibronectin‐rich, PDGFRα/β^+^ stromal cell network [103]. These TLS show prominent accumulation of B220^+^ B cells, class‐switched CD138^+^ plasma cells, and T peripheral helper (Tph)‐like PD‐1^+^CXCR5^−^ cells within the leptomeninges [103]. Interestingly, IgH^MOG^ B cells may also exacerbate Th17‐driven passive EAE in the NOD background through a mechanism dependent on IL‐23 [103], a well‐established encephalitogenic cytokine [124, 125].

### 4.3 Biozzi AB/H EAE

Immunization with spinal cord homogenate emulsified in CFA in young Biozzi ABH mice (8–12 weeks old) induces a relapsing–remitting form of EAE, which transitions into a chronic, nonremitting disease approximately 3 months after immunization [104, 126]. In contrast, applying the same immunization protocol to aged Biozzi ABH mice (12 months or older) leads to the immediate onset of a progressive, nonremitting disease course, bypassing the initial relapsing–remitting phase entirely [105]. Aged mice not only exhibit this altered disease trajectory but also demonstrate exacerbated neuropathology compared to their younger counterparts. This includes more pronounced axonal damage, heightened microglial activation, and a significant increase in CD3^+^ T cell infiltration into the spinal cord. Additionally, both the incidence of EAE and the rate of disease‐associated mortality rise with age. Notably, juvenile mice (younger than 2 weeks) display a remarkable resistance to EAE induction, suggesting a potential age‐dependent vulnerability to CNS autoimmune responses. While this model provides valuable insights into the relationship between aging, microglial activation, peripheral T cell infiltration, and WM pathology within the spinal cord, it does not reproduce the formation of leptomeningeal TLS near regions of cortical GM injury, as observed in people with PMS [15, 71, 126, 127, 128]. Furthermore, as with all active models of EAE, the autoimmune response in this model is triggered using spinal cord homogenate and CFA. It remains possible that some aspects of CNS pathology may be influenced or exacerbated by the strong adjuvant‐induced inflammatory response, potentially confounding the interpretation of disease mechanisms.

### 4.4 SJL/J PLP_139_ _‐151_ Active EAE

SJL/J mice are highly susceptible to both active and passive PLP_139‐151_‐driven EAE. Active immunization with PLP_139‐151_ in SJL/J mice induces a relapse‐remitting disease even without coadministration of pertussis toxin, making it a relevant model for understanding RRMS [106, 129]. On the other hand, adoptive transfer (A/T) of PLP_139‐151_‐primed T cells skewed ex vivo with IL‐23 induces a monophasic disease followed by a period of recovery in naïve recipient mice [43]. While disease course in SJL/J PLP_139‐151_ EAE can be influenced by method of EAE induction, sex [130] and age of mice [131, 132], and even substrain differences in gene copy numbers across vendors [107], the development of prominent leptomeningeal TLS in the brain remains consistent [43, 107, 132].

As mentioned, active immunization with PLP_139‐151_ induces a relapsing–remitting EAE (RR‐EAE) [13]. This disease course occurs with the appearance of TLS in the meninges and upregulation of *Baff* and *Cxcl13* transcripts that increase with each relapse and wane with each remission. During the first relapse, there is an influx of CD4^+^ T cells and some B cell accumulation in the leptomeninges. However, during remission, leptomeningeal B cells become more clustered, and T cells are still visible at similar levels throughout the disease course [13], suggesting that leptomeningeal TLS persists even as clinical symptoms subside. These observations support the notion that TLS‐derived factors may contribute towards progression over time, making this model an appealing tool for dissecting the roles of TLS that ultimately lead to disease progression.

MRI studies in mice have been pivotal in elucidating pathologies associated with neuroinflammation and protective or detrimental effects of therapeutic interventions. Calabresi and colleagues utilized the active SJL/J EAE model to better understand the relationship between MRI studies and corresponding histopathology [133]. They demonstrated that areas of leptomeningeal enhancement visible by MRI matched areas that showed high infiltration of immune cells, including areas with features consistent with TLS, such as an accumulation of FDCs, B cells, T cells, macrophages, and CXCL13‐producing cells [13, 133]. In adjacent cortical gray matter, they identified regions of damage, including demyelination, astrocytosis, and microgliosis. Utilizing the power of this model, Calabresi and colleagues showed that treatment with Bruton's tyrosine kinase (BTK) inhibitor reduced leptomeningeal enhancement in the treated mice compared to the vehicle group [133].

### 4.5 SJL/J PLP_139_ _‐151_ Passive EAE

The consistent induction of leptomeningeal TLS in the brain in SJL/J A/T EAE mice has established this model as a valuable tool for probing the well‐documented association between leptomeningeal inflammation and GM injury in MS [15, 69, 72, 134], a process that remains poorly understood. In the SJL/J A/T EAE model, recipient mice (8–10 weeks old) develop brain leptomeningeal TLS underpinned by a fibronectin^+^ and PDGFRα/β^+^ stromal cell network, exhibiting various degrees of lymphocyte infiltration across the leptomeninges overlying different anatomical locations in the brain, including the hippocampal, cerebellar, and brainstem sulci, and periventricular regions [43]. Notably, these TLS are associated with a gradient of cortical gray matter demyelination and increased microglial activation in TLS‐proximal regions [135]. While EAE and leptomeningeal TLS are initiated by Th17 cells in this model [43], the age of the recipient can alter the persistence of TLS. In aged recipient mice (> 8 months) that receive PLP_139‐151_‐primed Th17 cells from young donor animals, TLS is sustained in the brain leptomeninges, correlating with a progressive disease phenotype including loss of brain volume [132]. While CD4^+^ T cell numbers and cytokine secretion in the leptomeninges are relatively consistent between young and aged recipient mice, TLS in aged mice contained more neutrophils and class‐switched B cells [136]. This is also seen in postmortem brain tissues in people with PMS [132]. Using this A/T EAE model, we and others have been able to further interrogate the mechanisms of MS therapies on modulating leptomeningeal TLS and the role of aging in TLS persistence.

## 5 Formation and Persistence of Leptomeningeal TLS in MS/EAE

### 5.1 TLS Elaboration—The Lymphotoxin Pathway

As mentioned, LTβR signaling is critical for the formation of lymph nodes in utero and the homeostatic maintenance of secondary lymphoid tissue organization in the adult. Accordingly, the disruption of the LT pathway in mice via genetic deletion of *Lta*, *Ltb*, or *Ltbr* results in the absence of lymph nodes and Peyer's patches [4, 137], as well as architectural disorganization of the spleen and thymus [137]. Although LTαβ is expressed on LTi during development, in adults, LTαβ expression is found on T cells, B cells, innate lymphoid cells, and NK cells. Additionally, LIGHT (TNFSF14)—another ligand for LTβR—is expressed by T cells and myeloid cells, including neutrophils, macrophages, and DCs [4, 84].

It has been proposed that Th17 cells—a T helper subset implicated in MS and EAE—may mimic the in utero function of LTi cells to initiate TLS formation. In support of this, Pikor et al. adoptively transferred PLP_139–151_‐specific Th17 cells into SJL/J mice to induce EAE and examined the leptomeninges for TLS development [43]. They observed an expansion of gp38^+^CD31^−^PDGFRα^+^PDGFRβ^+^ stromal cells, characteristic of FRCs, in the leptomeninges of EAE mice. In vitro studies further demonstrated that the addition of recombinant IL‐17 and IL‐22 to meningeal fibroblasts could induce the expression of ECM components, such as fibronectin and collagen, implicating Th17‐derived cytokines in the earliest steps of fibroblast remodeling and the establishment of TLS. Immunofluorescence microscopy revealed the accumulation of CD4^+^ T cells and B220^+^ B cells within a fibronectin^+^ERTR7^+^ ECM network, forming organized structures. These TLS were situated near areas of myelin rarefaction, paralleling findings from postmortem MS tissue. To dissect the role of LTβR signaling, the authors administered LTβR‐Ig intrathecally to block LTβR signaling. While this did not significantly alter the number or spatial organization of gp38^+^ stromal cells, it led to a reduction in B220^+^ B cells within the TLS, suggesting that LTβR signaling contributes to the maintenance or recruitment of B cells within the meningeal niche. Further, meningeal fibroblasts expressed transcripts for the B cell‐attracting chemokine *Cxcl13*, and *Cxcl13* levels were nearly undetectable when EAE was induced in *Ltbr* ^−/−^ mice. Moreover, the absence of LTβR signaling in radio‐resistant, but not radio‐sensitive, cells impaired CD4^+^ T cell IL‐17 and IFN‐γ production.

Further work investigating the link between the LT pathway and TLS development is underway. In a preprint by Naouar et al., researchers used the SJL/J model to investigate whether inhibition of BTK impacts TLS formation and associated gray matter pathology [138]. Small molecule BTK inhibitors such as Tolebrutinib have been tested in PMS (HERCULES trial) and were found to slow disability progression compared to placebo [139]. Naouar and colleagues found that TLS development was abrogated in SJL/J A/T EAE mice following treatment with the BTK inhibitor remibrutinib. Further, treatment with remibrutinib protected against subpial cortical GM demyelination, microglial activation, and disruption of the glia limitans. By immunofluorescence, CXCL13 was found to be significantly reduced in EAE mice treated with remibrutinib within the leptomeninges. Whole tissue qPCR for lymphotoxin ligand transcripts revealed that remibrutinib‐treated mice downregulated leptomeningeal *Ltb* expression. These data suggest that BTK inhibition may influence the production of both CXCL13 and *Ltb*, and administration of an LTBR agonist to remibrutinib‐treated SJL/J A/T EAE mice reversed the protective effects of BTK inhibition and normalized CXCL13 expression in the leptomeninges to untreated levels [138]. Collectively, these results suggest a link between BTK, the LTBR pathway, and CXCL13 in the formation of brain leptomeningeal TLS and associated cortical pathology.

**TABLE 1:** Summary of EAE models featuring meningeal inflammation and/or gray matter demyelination.

Strain	Induction	Disease features	TLS location and composition
C57BL/6 [90]	Subcutaneous injection of MOG_35‐55_ with CFA supplemented with pertussis toxin	Monophasic EAE Spinal cord (white matter) demyelination	Limited/none, rather diffuse meningeal inflammation with T cells, B cells, neutrophils, and monocytes
C57BL/6 [96]	Adoptive transfer (intraperitoneal or intravenous) of MOG_35‐55_‐primed T cells	Monophasic EAE (young, < 6 months) Nonremitting EAE (old, > 8 months) Spinal cord (white matter) demyelination	Limited/none, rather, diffuse meningeal inflammation with T cells, B cells, neutrophils, and monocytes
C57BL/6 [97]	Introduction of heat‐killed *Mycobacterium* in the piriform cortex after immunization against MOG_35‐55_	Focal lesions in the brain, gray matter demyelination, microglia, and astrocyte activation adjacent to TLS	Delayed‐type hypersensitivity TLS, slow accumulation of T and B cells in the leptomeninges
C57BL/6, 2D2 T cells [98]	Spontaneous EAE driven by MOG‐reactive T cells, incidence increases with age and pertussis toxin	Monophasic EAE Spinal cord demyelination	Spinal cord meninges, clusters of T cells and B cells encapsulated by a reticulin^+^ ECM network
C57BL/6, IgH^MOG^ B cells [99]	Subcutaneous injection of MOG_35‐55_ with CFA	Chronic disease, nonremitting Spinal cord demyelination	Spinal cord meninges, large clusters of B cells
NOD [100, 101, 102]	Immunization with MOG_35‐55_ with CFA supplemented with pertussis toxin	RR‐EAE with transition to chronic disease	TLS in the spinal cord meninges with CD4^+^ T cells, B220^+^ B cells, and CD21/CD35^+^ follicular dendritic cells
NOD IgH^MOG^ [103]	Immunization with MOG_35‐55_ with CFA	Rapid and severe progressive EAE	Brain‐adjacent TLS with fibronectin‐rich, PDGFRα/β^+^ stromal cell network and prominent accumulation of B220^+^ B cells
Biozzi‐ABH [104, 105]	Immunization with spinal cord homogenate emulsified in CFA	RR‐EAE (young, < 3 months), then progressive after 3 months Severe, progressive EAE (old, > 12 months) Cortical demyelination, axonal/synapse loss adjacent to TLS	CD3^+^ T cell and B220^+^ B cell infiltration into the gray and white matter of the spinal cord, rather than the overlying leptomeninges. Microglia activation near regions of severe axonal damage. Damage tends to be perivascular
SJL/J [106, 107]	Subcutaneous injection of PLP_139‐151_ with CFA	RR‐EAE, cortical demyelination, axonal/synapse loss adjacent to TLS	Brain and spinal cord, B220^+^ B cells, CD4^+^ T cells, FDCs
SJL/J [43]	Adoptive transfer of PLP_139‐151_‐primed T cells	Monophasic EAE (young, < 6 months) Nonremitting EAE (old, > 8 months) Cortical demyelination, axonal/synapse loss adjacent to TLS	Brain and spinal cord, B220^+^ B cells, CD4^+^ T cells, CD11c^+^ myeloid cells

### 5.2 Expansion of the TLS: Th17 Cells and Neutrophils

**FIGURE 2:**
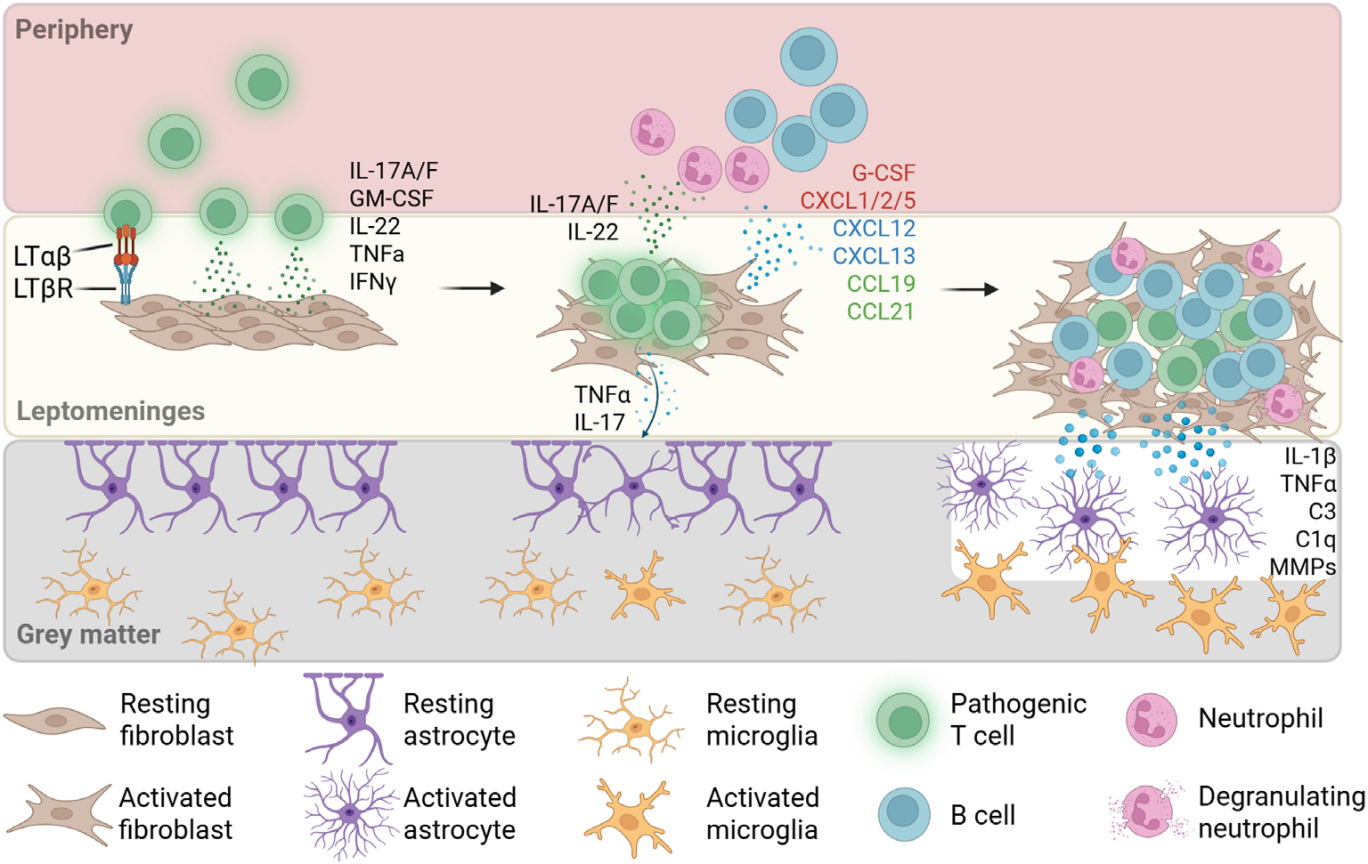
Elaboration of a leptomeningeal immune cell niche. (Left) Invasion of the leptomeninges by pathogenic Th17 cells. Engagement of LTβR on fibroblasts and secretion of inflammatory factors such as IL‐17 and IL‐22 induces fibroblast remodeling and activation. Glia limitans remains intact and gray matter is undisturbed. (Middle) Activated fibroblasts produce factors that induce granulopoiesis in the periphery (G‐CSF), as well as chemokines for neutrophil (in red), B cell (in blue), and T cell (in green) recruitment to the inflammatory milieu. Secretion of IL‐17 and TNFα by Th17 cells disrupts the subpial glia limitans. (Right) Prolonged inflammation and immune cell recruitment result in an elaborated lymphoid niche supported by activated fibroblasts (TLS). Inflammatory factors secreted by immune cells in the TLS further disrupt glia limitans (MMPs) and cause activation of glial cells (IL‐1β, TNFα, C3, C1q), resulting in damage to gray matter.

In addition to inducing the formation of TLS, Th17 cells contribute to meningeal inflammation by recruiting other immune cells, particularly (Table 1) neutrophils (Figure 2). Neutrophils can be recruited by Th17 cells either directly or indirectly. Activated Th17 cells secrete IL‐17A, which can induce stromal cells (endothelial cells, fibroblasts) and glia to produce neutrophil chemoattractants CXCL1, CXCL5, CXCL6, and CXCL8 [140, 141]. Additionally, IL‐17A induces upregulation of G‐CSF, which leads to increased production of granulocyte progenitor cells in the bone marrow, thereby modulating neutrophil granulopoiesis [142]. In a mouse model of *Staphylococcus aureus* infection, Cavagnero et al. demonstrated that IL‐17A promotes fibroblast‐mediated neutrophil recruitment. Using scRNA‐seq 1 day postinfection, they identified CXCL12^+^ fibroblasts as major producers of neutrophil chemokines (*Cxcl1, Cxcl2, Cxcl3, Cxcl5*), which were upregulated in response to IL‐17A [143]. Time‐course analysis showed early induction of *Cxcl1* and *Cxcl5* (within 3 h), followed by *Cxcl12* upregulation (by 12 h), with chemokine expression declining by Day 2 and returning to baseline by Day 10. The authors suggest *Cxcl1*/*Cxcl5* drive early neutrophil recruitment, while *Cxcl12* may mediate later recruitment. These findings raise the possibility that fibroblasts in the leptomeninges and brain may similarly coordinate neutrophil recruitment in IL‐17–driven neuroinflammation.

Given that neutrophil granules are packed with ECM‐degrading enzymes such as MMPs, neutrophils may further contribute to the remodeling of the leptomeningeal space, potentially to promote TLS expansion and/or to erode the underlying glia limitans and allow infiltration of other immune cells. Indeed, *Mmp2* and *Mmp9* double knockout mice show resistance to EAE and reduced leukocyte penetration of the CNS [144], as well as persistence of an intact BBB [144].

Lastly, a study by Harp et al. has established a putative role for neutrophils in guiding B cell trafficking to the spinal cord leptomeninges [145]. Using a model that ensures B cell‐restricted antigen presentation to T cells, these mice develop B cell‐rich follicles in the leptomeninges overlying the spinal cord. Blockade of CXCR2, a neutrophil‐specific chemokine receptor, resulted in the disappearance of B cell follicles, suggesting that neutrophil infiltration into the spinal cord leptomeninges is required for B cell recruitment. These data suggest that leptomeningeal neutrophil infiltration influences B cell recruitment. B cell infiltration into the CNS and the role of B cells in perpetuating the TLS will be discussed in the next section.

### 5.3 Expansion of the TLS: B Cells

Our group has recently profiled B cell subtypes that infiltrate the CNS during SJL/J A/T EAE, and the effect of age on the types of B cells that appear in the LM and dura [136]. While young mice have an accumulation of B cells in the leptomeninges at peak disease, old mice have a paucity of B cells [132]. Single‐cell RNA sequencing of these cells revealed that many of the B cells infiltrating the young LM are developing B cells based on the expression of *VpreB, Ebf1, Cd79b*, and *Ighm* [132]. These cells are partially resistant to anti‐CD20 treatment and may potentially be sustained by the increase in CNS BAFF concentrations after B cell depletion [136, 146]. Florescu et al. recently explored this phenotype further, finding that while young mice exhibit an accumulation of B220^low^ developing B cells that are IgM^+^ and IgD^+^, old mice harbored more class‐switched (IgM^−^IgD^−^) B220^high^ mature B cells [136]. Moreover, the ratio of B220^high^‐to‐B220^low^ B cells decreases with age [136]. Therefore, class‐switched B cells correlate with a nonremitting clinical phenotype and persistent LM aggregates, whereas B220^low^ B cells are associated with disease remission. In humans, aging is associated with a shift in B cell phenotype, including the expansion of age‐associated B cells (ABCs)—a subset implicated in chronic inflammation and autoimmunity [147]. Notably, ABCs accumulate within the dura mater over time and may displace other regulatory B cell subsets, such as IgA‐producing plasma cells, which have been shown by our group to deliver anti‐inflammatory cytokines like IL‐10 to the CNS during inflammation [148]. These findings suggest that the aging meningeal immune environment may influence B cell composition and function in ways that impact disease progression and regulation.

### 5.4 Regulation of the TLS: DCs, Monocytes and Macrophages

Monocytes, macrophages, and dendritic cells (DCs) may play important roles in CNS autoimmunity, but how they contribute to the formation and maintenance of TLS in the brain is unclear. Infiltrating monocytes are recruited to the CNS during inflammation via CCL2–CCR2 signaling and differentiate into macrophages within active lesions where they produce pro‐inflammatory cytokines (e.g., TNF, IL‐1β, IL‐6) and reactive oxygen species, contributing to demyelination and axonal damage [149, 150, 151]. These monocyte‐derived macrophages often adopt an M1‐like phenotype in active MS lesions, while a shift toward M2‐like profiles may be associated with tissue repair in resolving lesions [149, 152]. Macrophages and meningeal‐resident myeloid cells also play a structural and immunoregulatory role in TLS formation. They can interact with FRCs to remodel the ECM and produce lymphoid chemokines such as CXCL13, CCL19, and CCL21, which are critical for lymphocyte recruitment and organization within TLS [14, 153, 154, 155]. These chemokines attract CXCR5^+^ B cells and CCR7^+^ T cells, promoting compartmentalized adaptive immune responses. Notably, young SJL/J mice also have an accumulation of Ly6C^+^ monocytes in the leptomeninges during the acute phase of A/T EAE, although most of these monocytes disappear during remission. In aged mice, monocytes are continually present in the leptomeninges. Emerging studies have shown that monocytes from the skull and vertebral bone marrow can differentiate into macrophages (monocyte‐derived macrophages; MDMs) after infiltrating the brain parenchyma and brain borders [22, 24, 156]. These MDMs, although transcriptionally distinct from yolk‐sac derived microglia, can repopulate microglia‐depleted brains at steady‐state [24, 156], and invade the meninges and parenchyma during inflammation and injury [156]. Moreover, MDMs from the bone marrow were found to express genes associated with wound healing while blood‐derived myeloid cells expressed genes involved in propagating inflammation [24]. Follow‐up studies are required to determine whether the persistent monocyte signature in the leptomeninges of aged mice is pathogenic or immunoregulatory, and elucidating their origin may give some insights into their function.

Dendritic cells (DCs) are found within the leptomeninges, perivascular spaces, and SAS of both people with MS and EAE models, where they function as professional antigen‐presenting cells (APCs). They present CNS‐derived antigens to T cells via MHC class II and costimulatory molecules such as CD80/86 and CD40, supporting the activation and expansion of autoreactive T cells [157, 158]. Moreover, conventional DCs (cDCs) can produce IL‐12 and IL‐23, promoting the differentiation of Th1 and Th17 cells, both of which contribute to disease pathology and potentially to TLS induction [158, 159, 160, 161, 162]. Pikor et al. demonstrated the presence of leptomeningeal CD11c^+^ cells interspersed amongst CD4^+^ and B220^+^ cells in the SJL/J A/T model of EAE [43], although whether these myeloid cells were actively presenting antigen to lymphocytes is unknown. At steady‐state DCs have been found to populate brain regions where lesions tend to form, such as periventricular areas, and it has been theorized that these cells may act as gatekeepers for CD4^+^ T cells entering the brain [163, 164]. The Th17‐polarizing capability of DCs, coupled with their presence near or within TLS‐like structures, suggests a role for these cells in sustaining local immune activation [43, 157, 158, 165].

Collectively, myeloid cells may contribute to TLS persistence through chemokine‐driven recruitment of lymphocytes and polarization of Th17 differentiation while also driving pathogenic inflammation via antigen presentation and cytokine production.

### 5.5 Resolution of TLS

The resolution of TLS is a complex and poorly understood process, especially in the context of chronic diseases like MS, where we only have cross‐sectional (postmortem) tissue to study their composition. In peripheral tissues, TLS resolution is thought to occur via a combination of mechanisms, including the cessation of pro‐inflammatory cytokine and chemokine signaling, loss of stromal cell activation, and re‐entry of immune cells into circulation or draining lymphatics. Anti‐inflammatory cytokines (e.g., IL‐10 [121, 148], TGF‐β), regulatory T cells [166, 167], and immunosuppressive macrophages and B cells or plasma cells [60] may also play active roles in suppressing lymphoid neogenesis and promoting tissue remodeling [167].

Aging appears to negatively impact the resolution of TLS. As mentioned earlier, SJL/J mice over 8 months of age develop TLS following adoptive transfer of encephalitogenic T cells, and unlike young mice, these TLS persist in the CNS concomitant with a nonremitting clinical phenotype. This suggests that age is a predictor of impaired TLS resolution. One possible reason that has been explored in other settings is due to dysfunctional clearance mechanisms in the aged brain. For many years, the CNS was thought to lack classical lymphatic drainage. However, recent discoveries have overturned this view, revealing that the brain has both lymphatic and glymphatic mechanisms for fluid and solute clearance. Studies investigating lymphatic drainage have shown that this could be mediated by lymphocytes egressing from the leptomeninges through arachnoid cuff exit (ACE) points that lead into the dura, as molecules for retention are slowly outweighed by chemotactic signals from elsewhere in the periphery, such as the dura or cervical lymph nodes.

Rustenhoven and colleagues recently found that an aged dural lymphatic system exhibits changes in ECM remodeling [168]. For example, using immunostaining for type I collagen fibers, they found thicker bands of collagen‐positive staining near the dural sinuses of aged mice compared to young. This was supported by an increase in *Col1a1* expression in the aged dura compared to the young dura. Furthermore, ECM remodeling driven by constitutive expression of *Tgfbr1* using an adeno‐associated viral (AAV) vector resulted in an impaired drainage of intrathecal OVA to the cervical lymph nodes as well as impaired accumulation of OVA in dural macrophages and DCs. These data suggest ECM remodeling results in impaired clearance of CSF proteins, as well as a deficit in immunosurveillance by dural myeloid cells. Other studies in the context of subarachnoid hemorrhage and Alzheimer's disease have shown that promoting T cell egress via the CCR7‐CCL21 pathway is crucial for neuroprotection [169, 170]. It is tempting to speculate that impaired lymphatic drainage due to ECM remodeling may also cause lymphocytes to become trapped in the leptomeninges during neuroinflammation and may act as a roadblock to resolving inflammation in aged SJL/J A/T EAE mice.

## 6 Concluding Thoughts

Much progress has been made in our understanding of how TLS forms in brain‐adjacent regions, specifically the leptomeninges. This has tremendous impact not only on how we think about the neuro‐immune axis but also on how we may treat PMS, which is characterized by persistent leptomeningeal TLS that correlates with GM pathology. However, many key questions remain unresolved. For example, what is happening in the dura that overlies leptomeningeal TLS? Of note, Florescu et al. compared the kinetics of B cell infiltration into the brain, leptomeninges, and dura during neuroinflammation and observed a conspicuous loss of immature dural B cells during EAE [136] followed by their accumulation in the leptomeninges and brain parenchyma in young but not old mice. Whether these immature dural B cells contribute to disease remission remains to be determined. Another black box is the identity of leptomeningeal fibroblasts and determining if they change during steady state versus during MS/EAE [171]. Do fibroblasts perpetuate neuroinflammation by creating a self‐sufficient niche in the CNS? In addition, we know that aging alters fibroblast phenotype in the context of traumatic brain injury [172, 173]; is the same true for EAE, and if so, could that be another reason why TLS does not resolve in aged mice? Answers to these questions will allow us to better understand the dynamics of TLS formation and resolution and may provide a therapeutic window of opportunity for the treatment of PMS.

## Author Contributions

M.Z., A.A.W., and J.L.G. worked together in writing the manuscript. M.Z. compiled the sections and generated figures. M.Z., A.A.W., and J.L.G. reviewed and edited the study.

## Conflicts of Interest

The authors declare no conflicts of interest.

## Data Availability

Data sharing not applicable to this article as no datasets were generated or analysed during the current study.
